# DCIR suppresses osteoclastic proliferation and resorption by downregulating M-CSF and RANKL signaling

**DOI:** 10.3389/fimmu.2023.1159058

**Published:** 2023-05-17

**Authors:** Tomonori Kaifu, Takumi Maruhashi, Soo-Hyun Chung, Kenji Shimizu, Akira Nakamura, Yoichiro Iwakura

**Affiliations:** ^1^ Division of Immunology, Faculty of Medicine, Tohoku Medical and Pharmaceutical University, Sendai, Miyagi, Japan; ^2^ Laboratory of Molecular Immunology, Institute for Quantitative Biosciences, The University of Tokyo, Tokyo, Japan; ^3^ Center for Animal Disease Models, Research Institution for Biological Sciences, Tokyo University of Science, Noda, Chiba, Japan

**Keywords:** C-type lectin receptor, osteoclast, cytokines, oligosaccharides, homeostasis, metabolism, DCIR

## Abstract

Dendritic cell immunoreceptor (DCIR) is an inhibitory C-type lectin receptor that acts as a negative regulator in the immune system and bone metabolism. We previously revealed that DCIR deficiency enhanced osteoclastogenesis and antigen presentation of dendritic cells, and that asialo-biantennary N-glycan (NA2) functions as a ligand for DCIR. NA2 binding to DCIR suppressed murine and human osteoclastogenesis that occurs in the presence of M-CSF and RANKL. The DCIR-NA2 axis, therefore, plays an important role in regulating osteoclastogenesis in both mice and humans, although the underlying mechanisms remain unclear. Here we found that *Dcir* ^−/−^ bone marrow–derived macrophages (BMMs) exhibited greater proliferative and differentiation responses to M-CSF and RANKL, respectively, than wild-type (WT) BMMs. Moreover, *Dcir* ^−/−^ osteoclasts (OCs) increased resorptive activity and cell fusion more significantly than WT OCs. DCIR deficiency affects gene expression patterns in OCs, and we found that the expression of neuraminidase 4 was increased in *Dcir* ^−/−^ OCs. Furthermore, DCIR-NA2 interaction in WT BMMs, but not *Dcir* ^−/−^ BMMs, decreased Akt phosphorylation in response to M-CSF and RANKL. These data suggest that DCIR regulates osteoclastogenesis by downregulating M-CSF and RANKL signaling, and that DCIR-mediated signaling may contribute to the terminal modification of oligosaccharides by controlling the expression of glycosylation enzymes.

## Introduction

Osteoclasts (OCs) are hematopoietic cell–derived bone cells that have a unique ability to absorb bone minerals and are responsible for continuous remodeling of bone structure throughout life. OCs are differentiated from myeloid progenitor cells in the presence of macrophage colony stimulation factor (M-CSF), which promotes proliferation, survival, and mobility of monocyte/macrophage lineage cells, and receptor activator of nuclear factor-κB ligand (RANKL), which is responsible for gene expression of osteoclastogenesis (1). The binding of M-CSF to its receptor, CSF-1R, initiates signaling pathways such as those related to PLC-γ and Akt, and DAP12, a signal adaptor molecule, is indispensable for M-CSF–induced proliferation and survival (2). RANKL binding to its receptor, RANK, transduces a signal *via* TRAF6 and activates the NF-κB and MAPK pathways, leading to induction of expression of NFATc1, the master regulator of osteoclastogenesis (3). NFATc1 and other transcription molecules, including PU.1 and AP-1, promote the expression of osteoclastogenic genes.

Receptors originating from immunological cells interact with signaling pathways downstream of RANK. The induction of NFATc1 is strengthened by the calcium signal that is regulated by DAP12 and FcRγ, immunoreceptor tyrosine-based activation motif (ITAM)-harboring molecules that associate with immunoglobulin-like receptors (TREM2, SIRPβ1, OSCAR, PIR-A, and FcγRIII) and C-type lectin receptors (CLRs) (Siglec15 and Mincle) (4–6). Inhibitory signals mediated by the tyrosine phosphatases SHP-1 and SHP-2, which are recruited *via* an immunoreceptor tyrosine-based inhibitory motif (ITIM), counter-regulate signals downstream of the ITAM-harboring receptors in the immune system. Recent studies showed that ITIM-harboring receptors (PIR-B, gp49B, SIRPα, PECAM-1, and CLM-1) negatively regulated OC development and functions (7–11). Moreover, Maruhashi et al. revealed that dendritic cell immunoreceptor (DCIR), an inhibitory CLR, maintains bone homeostasis, and that *Dcir* ^−/−^ mice spontaneously developed joint ankylosis with age (12). Although ITIM-harboring immunoreceptors are critical regulators that determine the thresholds of cell activities and prevent their overactivation, the physiological functions of these immunoreceptors in OCs remain to be clarified.

DCIR is a unique CLR characterized by a canonical ITIM in its cytoplasmic portion (13) and by inhibitory functions mediated by ITIM-transduced inhibitory signals (14). *Dcir* ^−/−^ mice spontaneously develop autoimmune sialadenitis and enthesitis and exhibit increased susceptibility to animal models of autoimmune diseases such as rheumatoid arthritis and multiple sclerosis (15, 16). Aged *Dcir* ^−/−^ mice show bone abnormalities, including fibrocartilage proliferation and heterotopic ossification in joints, due to the expansion of IFNγ-producing T cells (12, 17), indicating that DCIR plays an important role in negatively regulating not only bone metabolism, but also the immune system. In general, a ligand must bind to a receptor in order to induce the receptor’s functions, and we have identified asialo-biantennary N-glycan (NA2) as a DCIR ligand. Neuraminidases remove the terminal sialic acid residue from oligosaccharides and expose NA2. When the neuraminidase derived from *Arthrobacter ureafaciens* was administered to mice with collagen-induced arthritis, it improved their clinical score and ameliorated the bone damage in the ankle joints in a DCIR-dependent manner. DCIR is expressed in OCs in addition to dendritic cells (DCs) and neutrophils, and DCIR deficiency enhanced osteoclastogenesis in the presence of M-CSF and RANKL. Furthermore, DCIR–NA2 interaction suppressed osteoclastogenesis (18). However, the signaling pathway underlying DCIR control of OC differentiation and functions remains to be elucidated.

In this study, we examined this issue and showed that *Dcir* ^−/−^ bone marrow–derived macrophages (BMMs) were more responsive to M-CSF and RANKL than WT BMMs. *Dcir* ^−/−^ BMMs showed greater proliferation than WT BMMs in response to M-CSF, and DCIR deficiency enhanced osteoclastogenesis in response to RANKL. In addition, while *Dcir* ^−/−^ OCs exhibited increased resorption activity and cell fusion compared to WT OCs, the two cell types showed comparable formation of actin rings, a characteristic structure of OCs responsible for bone resorption. DCIR deficiency affected gene expression patterns in OCs, and the gene sets associated with biosynthetic and metabolic processes were increased in *Dcir* ^−/−^ OCs. DCIR binding to NA2 suppressed Akt phosphorylation downstream of MCSF and RANKL signaling. These observations suggest that DCIR is a unique receptor that negatively regulates M-CSF and RANKL signaling by binding to NA2, and that the DCIR–NA2 axis is a potential target in bone metabolic diseases.

## Results

### *Dcir* ^−/−^ BMMs are hyperresponsive to M-CSF and RANKL

**Figure 1:**
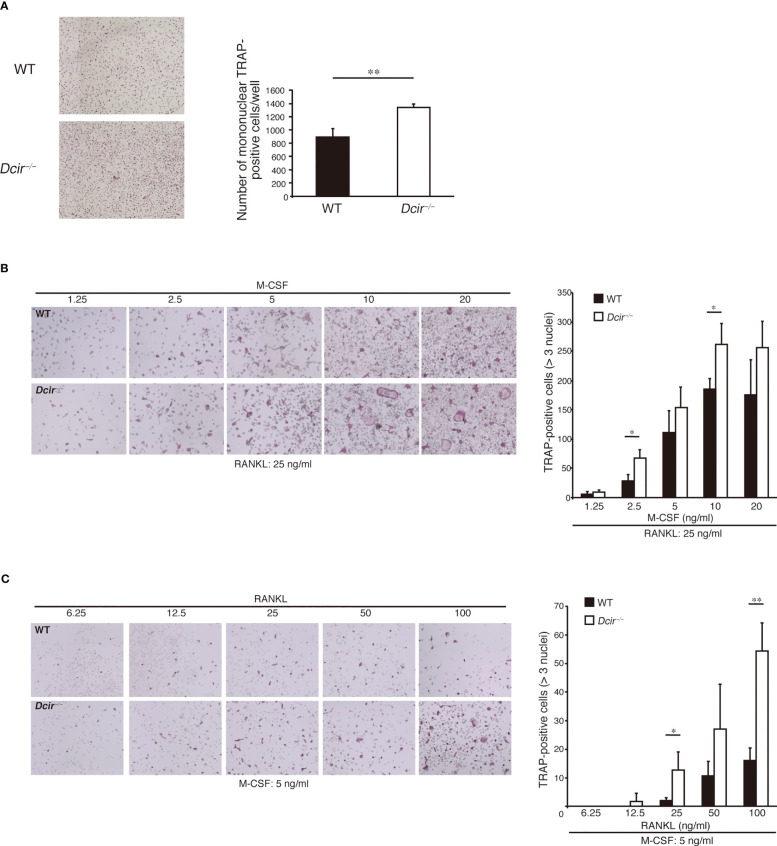
DCIR-deficient osteoclast precursor cells are hyper-responsive to M-CSF and RANKL. **(A)**. TRAP+ MNC differentiation from BMMs was induced by incubation with M-CSF (20 ng/ml) and RANKL (100 ng/ml), and inspected on day 2 just before the appearance of multinuclear cells. Objective 4×. **(B, C)** TRAP+ OC differentiation from non-adherent BMCs was examined in the presence of the indicated concentrations of M-CSF and a low concentration of RANKL (25 ng/ml) **(B)**, or the indicated concentrations of RANKL and a low concentration of M-CSF (5 ng/ml) **(C)**. Objective 4×. The data are representative of three independent experiments. The error bars present means ± standard deviation (s.d) of triplicate cultures. *P<0.05, **P<0.01.

**Figure 2:**
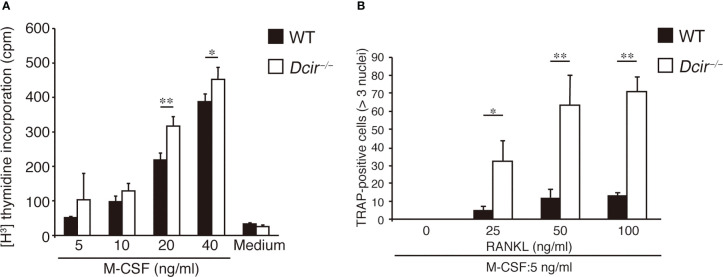
Osteoclast proliferation and differentiation are increased in response to M-CSF and RANKL. **(A)** BMMs (300,000 cells per well) were incubated at the indicated concentrations of M-CSF for 2–3 h in 96-well plates, and [3H] thymidine incorporation was examined for 48 h. **(B)** OC differentiation from BMMs (500,000 cells) was examined in the presence of various concentrations of RANKL and a low concentration of M-CSF (5 ng/ml) for 3 days. The data are representative of two independent experiments. The error bars present means ± s.d of triplicate cultures. *P<0.05, **P<0.01.

We previously found that DCIR is expressed in macrophages and OCs, and its deficiency resulted in enhanced osteoclastogenesis in the presence of M-CSF and RANKL (18). To understand how *Dcir* ^−/−^ BMMs increase OC formation, we examined the *in vitro* differentiation of tartrate-resistant acid phosphatase (TRAP)-positive mononuclear cells (MNCs), which are destined to differentiate into OCs just before they fuse with each other. Upon incubation of BMCs with M-CSF for 4 days, the number of TRAP^+^ *Dcir* ^−/−^ MNCs was significantly increased compared with that of WT MNCs (**Figure 1A**). To examine the responsiveness of *Dcir* ^−/−^ BMMs to M-CSF and RANKL, we analyzed osteoclastogenesis in WT and *Dcir* ^−/−^ BMMs by increasing M-CSF concentrations in the presence of low concentrations of RANKL, and vice versa. *Dcir* ^−/−^ BMCs differentiated into TRAP^+^ multinucleated cells more efficiently than WT BMCs (**Figures 1B, C**), suggesting that DCIR deficiency causes BMMs hyperresponsivity to M-CSF and RANKL. Consistent with this, *Dcir* ^−/−^ BMMs exhibited a stronger proliferative response to M-CSF than WT BMMs (**Figure 2A**), indicating that DCIR-mediated signaling downregulates M-CSF–induced proliferation. Although M-CSF is involved in the survival of OCs as well as in their proliferation (19), OC apoptosis were not affected by DCIR deficiency (**Figure S1A**). Furthermore, *Dcir* ^−/−^ BMMs differentiated into multinucleated OCs much more efficiently than WT BMMs, even under low concentrations of RANKL (25 and 50 ng/ml) (**Figure 2B**). Thus, both M-CSF–induced TRAP^+^ MNC proliferation and RANKL-induced multinucleated OC differentiation were enhanced in *Dcir* ^−/−^ BMMs.

### DCIR deficiency does not affect the development of OC precursors

OCs are formed by the fusion of monocyte lineage cells that originate from hematopoietic cell populations in bone marrow (BM) and peripheral blood. We examined whether DCIR–NA2 interaction affected the development of OC precursors in BM. We determined the frequency of OC precursors with flow cytometer. The frequency of CD11b^lo^Ly6C^hi^ OC precursors (20) in *Dcir* ^−/−^ BMCs was comparable with that of WT mice (**Figures S2A, B**). Moreover, we examined the RANK (CD265)– and c-fms (CD115)–expressing OC precursors in BMC (21) and there were no differences of CD265+cells, CD265+CD115+cells, and CD115+cells between WT and *Dcir* ^−/−^ BMCs (**Figures S2C, D**). Furthermore, we evaluated the frequency of OC precursor populations expressing c-kit (CD117) and CD115 (22). In CD11b^-/low^B220^-^CD3^-^ (triple negative) cells, the percentage of CD117^hi^CD115+cells, CD117^int^CD115+cells, and CD117^low^CD115+cells were comparable between WT and *Dcir* ^−/−^ BMCs (**Figures S2E, F**). These data suggest that DCIR is not involved in the differentiation of these precursors.

### DCIR deficiency enhances OC resorptive activity and cell fusion

**Figure 3:**
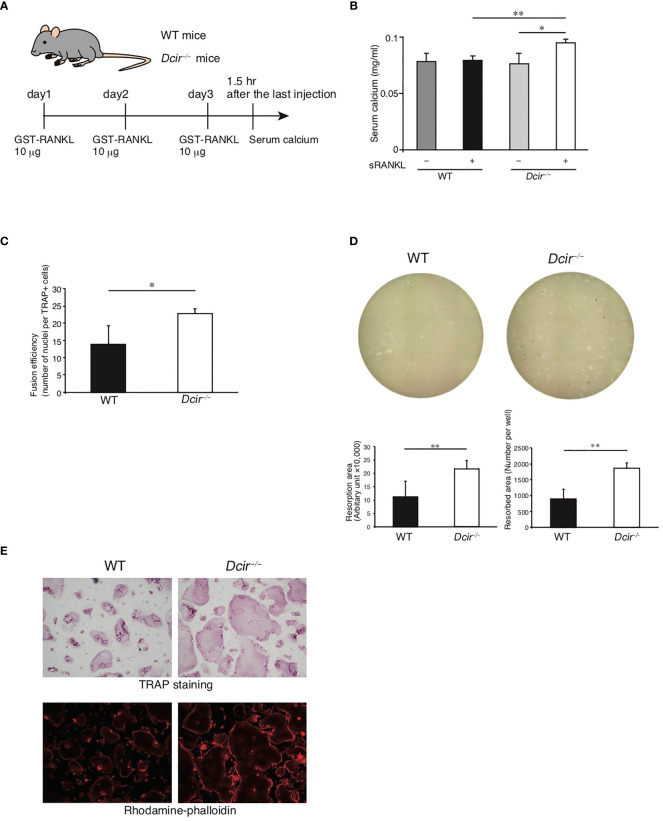
Resorption activity and cell fusion are increased in *Dcir* ^−/−^ osteoclasts but actin-ring formation activity is unchanged. **(A)** Experimental design of RANKL-induced bone destruction for evaluating in vivo DCIR deficiency. PBS or GST-RANKL were intraperitoneally administered for 3 days and mice were sacrificed 90 min after the last administration. **(B)** Serum concentrations of calcium ions in RANKL-injected mice. Serum concentrations of calcium ions were determined after the third intraperitoneal injection of RANKL or PBS [WT; GST-RANKL (n=4) and PBS (n=4), *Dcir* ^−/−^ mice; GST-RANKL (n=4) and PBS (n=4)]. Data are representative of two independent experiments. Statistical significance was evaluated by one-way ANOVA with Dunnett’s post hoc test (**P<0.01; *P<0.05). **(C)** WT and *Dcir* ^−/−^ BMMs were induced to form OCs by M-CSF and RANKL, followed by staining with TRAP and hematoxylin for counting OCs and nuclei, respectively. The fusion efficiency value is defined as the number of nuclei divided by the number of OCs. The bars represent the means ± s.d. of triplicate cultures. *P<0.05. **(D)** Pit formation assay in an Osteo Assay Plate. The images of the Osteo Assay Plate well were taken at objective 4×. Bars represent the means ± s.d. of triplicate cultures. **P<0.01. **(E)** OC formation was assessed by TRAP staining, and the actin structure of OCs was detected using rhodamine phalloidin. Images of rhodamine phalloidin were acquired by fluorescence microscopy. Data are representative of three independent experiments **(B–D)**.

We previously revealed that the number of OCs in the distal metaphysis of the tibia was increased in *Dcir* ^−/−^ mice and the *in vitro* induction of OCs was enhanced in *Dcir* ^−/−^ BMMs compared to WT (12, 18). Therefore, we studied the roles of DCIR in OC differentiation and resorptive activity using a mouse model of osteoporosis involving intraperitoneal injection of modified RANKL (glutathione-S-transferase (GST)-fused RANKL) (**Figure 3A**). In this model, systemic bone loss is induced by an increase in the number of OCs (23). We injected 10 μg of GST-RANKL into each mouse in order to induce minimum osteoclastic responses in WT mice. As shown in **Figure 3B**, *Dcir* ^−/−^ mice exhibited significantly increased serum calcium levels after GST-RANKL administration, while this increase was not observed in WT mice, indicating that DCIR negatively regulates RANKL-mediated osteoclastogenesis *in vivo*. Moreover, *Dcir* ^−/−^ TRAP^+^ cells contained more nuclei per cell than WT TRAP^+^ cells after induction of OC differentiation with M-CSF and RANKL (**Figure 3C**), indicating increased fusion activity of *Dcir* ^−/−^ BMMs. The number of pits formed by OCs on inorganic crystalline calcium phosphate and the sizes of the resorption areas were significantly increased in *Dcir* ^−/−^ OCs (**Figure 3D**). By contrast, DCIR deficiency had only a marginal effect, if any, on the formation of actin rings, a structure required for OC resorption activity (**Figure 3E**). These data suggest that DCIR is directly involved in osteoclastic activities such as bone resorption and cell–cell fusion.

### DCIR deficiency affects the gene expression pattern in OCs

**Figure 4:**
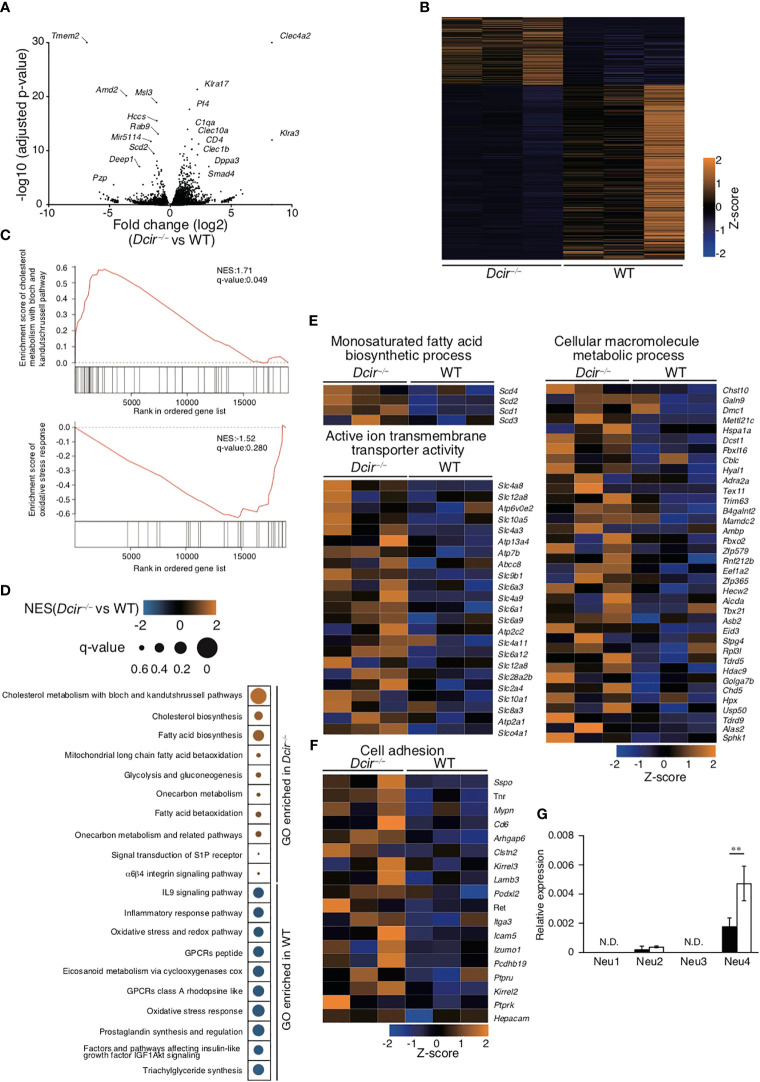
DCIR deficiency changes osteoclast gene expression. **(A)** Changes in gene expression were examined between WT (n=3) and *Dcir* ^−/−^ (n=3) OCs, and −log10 P values versus log2 fold change are plotted on the y and x axes, respectively. Genes with high fold changes are labeled. Genes (Tmem72 and Clec4a2) with a −log10 P value greater than 30 are shown with a value of 30 in the volcano plot. **(B)** Heatmap of changes in gene expression between WT (n=3) and *Dcir* ^−/−^ OCs (n=3). One thousand genes with a high −log10-adjusted P value were selected. **(C, D)** GSEA was conducted to provide insights into biological pathways by searching gene sets in WT and *Dcir* ^−/−^ OCs. Enrichment scores of cholesterol metabolism with the bloch and kandutshrussell pathways in *Dcir* ^−/−^ OCs, and of oxidative stress responses in WT OCs, are shown in **(C)** The normalized enrichment score (NES) and q-value of the indicated gene sets are presented using color- and size-based scales. Orange color and small size indicate high values, and blue color and large size indicate low values. **(E)** Changes in gene expression in the indicated categories associated with biosynthetic and metabolic processes and ion transmembrane transport. The gene changes are shown using a scale from high (orange) to low (blue). **(F)** Genes associated with cell adhesion are shown using a color scale from high (orange) to low (blue). **(G)** Gene expression of neuraminidases in BMMs and OCs. mRNA was collected from OCs induced from BMMs in the presence of M-CSF and RANKL. Neuraminidase-specific mRNAs were quantified by qPCR. The quantity was normalized to Gapdh. The data are representative of three independent experiments **(E)**. The error bars present means ± s.d of triplicate wells. **P<0.01. N.D. stands for "not detected".

To further examine the effect of DCIR deficiency on osteoclastogenesis, we analyzed the transcriptional profiles of OCs that underwent 4 days of culture in the presence of RANKL. The expression of 121 genes in *Dcir* ^−/−^ OCs was significantly upregulated compared with WT OCs, while that of 431 genes was remarkably downregulated (**Figures 4A, B**). *Dcir* ^−/−^ OCs showed increased expression of osteoclastogenic genes upregulated by RANKL stimulation, such as *Ctsk*, *Acp5*, and *Nfatc1* (**Figure S3A**). Gene ontology (GO) analysis of the gene expression in *Dcir* ^−/−^ DCs showed that DCIR deficiency upregulated the gene sets associated both with antigen presentation and with T cell proliferation, cytokine activity, and receptor signaling (18). However, WT and *Dcir* ^−/−^ OCs exhibited a smaller number of gene sets involved in these DC functions, and *Dcir* ^−/−^ OCs tended to demonstrate fewer such gene sets compared to WT OCs, meaning that DCIR deficiency facilitates differentiation into OCs (**Figure S3B**). To understand the biological characteristics of genes whose expression changes as a result of DCIR deficiency, we conducted gene set enrichment analysis (GSEA) in WT and *Dcir* ^−/−^ OCs. We identified diverse gene sets, including those involved in cholesterol biosynthesis and fatty acid biosynthesis in *Dcir* ^−/−^ OCs, and those related to the oxidative stress response and the IL-9 signaling pathway in WT OCs (**Figures 4C, D**). GO analysis highlighted biosynthetic and metabolic processes and ion transmembrane transporters in the transcriptional signatures of *Dcir* ^−/−^ OCs (**Figure 4E**). In addition, we observed higher expression of genes associated with cell adhesion in *Dcir* ^−/−^ OCs than in WT OCs (**Figure 4F**, which is consistent with an earlier study showing that M-CSF is involved in macrophage adhesion (2).

We previously identified asialo-biantennary N-glycan (NA2) as a functional ligand for DCIR (18). Neuraminidases catalyze the removal of the terminal sialic acid residue from oligosaccharides, while sialyltransferases transfer sialic acids to the terminal sites of oligosaccharides. The terminal sialyl modification in sugar chains is controlled by the balance between neuraminidases and sialyltransferases; however, RNA sequencing (RNA-seq) in this study did not reveal any obvious differences in the gene expression of neuraminidases or sialyltransferases (**Figure S3C**). To further confirm the gene expression patterns of neuraminidases in OCs, we performed quantitative PCR (qPCR) analysis of Neu1, Neu2, Neu3, and Neu4. In accordance with RNA-seq analysis, Neu1 and Neu3 showed only marginal gene expression with no significant differences between *Dcir* ^−/−^and WT OCs. Neu2 and Neu4 also had low expression, but that of Neu4 was higher in *Dcir* ^−/−^ OCs than in WT OCs (**Figure 4G**). Thus, these data suggest that DCIR-mediated signaling may be partially involved in the regulation of neuraminidase expression in OCs.

### Interaction between DCIR and its ligand downregulates M-CSF and RANKL signaling and OC activity

**Figure 5:**
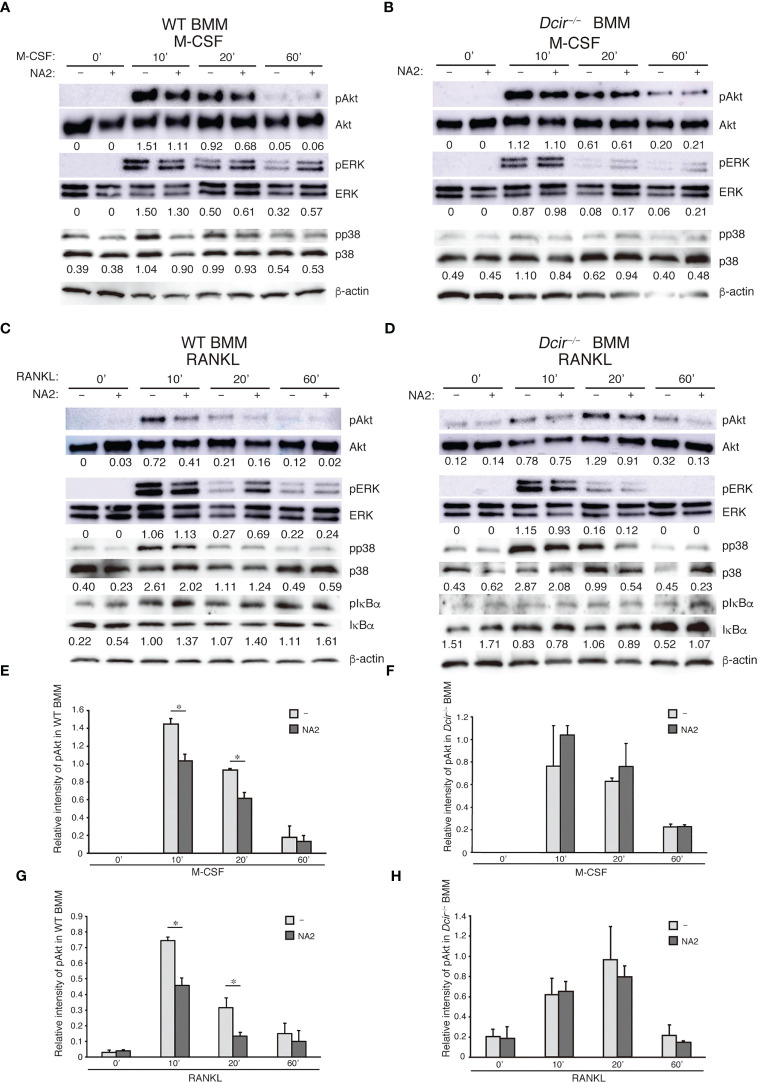
DCIR–NA2 interaction downregulates M-CSF– and RANKL-mediated signaling and reduced osteoclast activity. The effect of NA2 treatment on M-CSF– and RANKL–mediated signaling. WT and *Dcir* ^−/−^ BMMs were pre-treated with NA2 (1 μg/ml) for 6 h and then stimulated with M-CSF **(A, C)** or RANKL **(B, D)** for the indicated periods in the presence of NA2. Phosphorylation of each protein kinase was detected by western blotting using a specific antibody against kinases (**A**, **B**; WT BMMs, **C**, **D**; *Dcir* ^−/−^ BMMs). The data are representative of at least two independent experiments **(A–D)**. The values described below images are relative quantity of phosphorylated kinase protein. **(E, F)** Relative intensity of phosphorylated Akt in BMMs and *Dcir* ^−/−^ BMMs after M-CSF stimulation. **(G, H)** Relative intensity of phosphorylated Akt in WT and *Dcir* ^−/−^ BMMs after RANKL stimulation. Light gray columns show NA2–untreated BMMs and dark gray columns are NA2-treated BMMs. Bars represent the means ± s.e.m. of two independent experiments. *P<0.05 **(E–G)**.

DCIR deficiency increased OC responsiveness to M-CSF and RANKL, systemic bone loss induced by soluble RANKL administration, bone resorption activity, and cell–cell fusion in the presence of M-CSF and RANKL. These data suggest that DCIR-mediated signals may be involved in regulating the downstream signaling of M-CSF and RANKL. We found no differences in the phosphorylation levels of various kinases in WT and *Dcir* ^−/−^ BMMs upon stimulation with M-CSF or RANKL (data not shown). We previously showed that some macrophages and OCs were positive for the DCIR ligand in the absence of neuraminidase treatment (18), indicating that the amount of existing ligand for DCIR in BMMs may be insufficient to induce detectable DCIR-mediated inhibitory signaling. To strengthen this signaling, we pre-treated BMMs with NA2, a functional ligand for DCIR, in serum-free medium, then further stimulated the cells with either M-CSF or RANKL. We found that both M-CSF and RANKL treatment increased Akt phosphorylation in BMMs, while NA2 treatment decreased Akt phosphorylation in WT BMMs (**Figures 5A–G**) but not in Dcir−/− BMMs (**Figures 5B–H**). NA2 had no effect on MAPKs or IκBα upon M-CSF or RANKL stimulation in WT BMMs (**Figures 5A–C**). These findings suggest that the interaction between DCIR and NA2 regulates osteoclastogenesis by suppressing M-CSF and RANKL signaling.

## Discussion

We previously showed that aged *Dcir* ^−/−^ mice spontaneously developed joint ankylosis, and that this and other bone abnormalities were due to an increased number of IFNγ-producing T cells (12). Moreover, DCIR deficiency enhanced OC differentiation from BMMs in the presence of M-CSF and RANKL and facilitated osteoclastogenesis in a co-culture system involving BM cells and osteoblasts (18). In addition to indirectly regulating osteoclastogenesis *via* IFNγ produced by T cells, DCIR directly regulates OC formation through ITIM-mediated signaling. In this study, we showed that DCIR-mediated signaling downregulated OC responsiveness to M-CSF and RANKL. DCIR deficiency affected the gene expression pattern in OCs, with up-regulation of gene sets associated with metabolic processes. Moreover, Akt phosphorylation in BMMs, which was induced by stimulation with M-CSF and RANKL, was reduced by NA2 treatment in a DCIR-specific manner.

M-CSF regulates OCs by promoting differentiation, proliferation, cytoskeletal reorganization, and motility. M-CSF binding to the M-CSF receptor (CSF-1R) results in CSF-1R dimerization and induces autophosphorylation of tyrosine residues in the cytoplasmic region, leading to activation of signaling molecules. Tyrosine 721 phosphorylation in the cytoplasmic domain of CSF-1R is a docking site for phosphatidylinositol 3-kinase, which activates Akt signaling pathways involved in proliferation and anti-apoptosis (24). We found that DCIR deficiency increased the number of mononuclear TRAP^+^ cells, and [H^3^] thymidine incorporation in WT BMMs and NA2 treatment reduced Akt phosphorylation in response to M-CSF. The tyrosine phosphatases SHP-1 and SHP-2, which are recruited to the phosphorylated sites of DCIR, are responsible for DCIR-mediated signaling, and therefore it is unlikely that Akt, a serine/threonine kinase, is the substrate of these phosphatases. One of the potential substrates of phosphatases that are functionally activated by DCIR is therefore tyrosine 721 of MCSFR. Tyrosine phosphorylation of MCSFR was elevated in BMMs from SHP-1–deficient *motheaten* mice, suggesting that SHP-1 is involved in regulating MSCFR dephosphorylation. However, deletion of macrophage- and monocyte-specific SHP-1 by LysM-cre did not result in an abnormal phenotype (25, 26). The other potential substrate is DAP12, an adaptor signaling molecule required for M-CSF–induced macrophage proliferation and survival. Phosphorylated ITAM of DAP12 serves as a docking site for the tyrosine kinase Syk, a molecule that is important for M-CSF–mediated proliferation and survival (27). DAP12 deficiency impaired M-CSF–mediated proliferation and survival, whereas M-CSF–induced Akt phosphorylation was unchanged in *Dap12* ^-/-^ BMMs (2). However, the signaling networks downstream of CSF-1R are still being investigated, and further research is necessary to determine how DCIR-mediated signaling regulates potential target receptors through phosphatase activity.

RANKL plays an essential role in OC differentiation. RANKL binding to its receptor, RANK, induces oligomerization of RANK and recruits TRAF6, leading to activation of the MAPK and NF-κB pathways. NA2 treatment did not affect RANKL-induced phosphorylation in the MAPK or NF-κB pathways in WT or *Dcir* ^−/−^ BMMs, although it did reduce Akt phosphorylation in WT BMMs but not *Dcir* ^−/−^ BMMs. The PI3K/Akt pathway is involved in RANKL-induced OC differentiation (28, 29). SHP-1 downregulates OC differentiation by inhibiting the PI3K/Akt signaling cascade through interaction with TRAF6 (30, 31). An ITIM-harboring immunoglobulin-like receptor, gp49B, negatively regulates osteoclastogenesis by inducing the association of SHP-1 with TRAF6 (11). DCIR-activated SHP-1 might form a complex with RANK and TRAF6 and reduce RANKL-induced Akt phosphorylation, although the exact substrate of SHP-1 in the DCIR-mediated regulation of osteoclastogenesis remains to be elucidated. In addition, the RANKL–RANK axis activates NFATc1 by initiating calcium ion–dependent signaling through cooperation with immunoreceptors associated with ITAM-harboring signaling molecules (32). DAP12 deficiency diminished multinuclear OC formation following *in vitro* induction with M-CSF and RANKL, and regarding Siglec15, a CLR associated with DAP12, RANKL-induced activation of the PI3K/Akt pathway was dampened in *Siglec15* ^−/−^ cells (5, 33). Similarly to M-CSF–induced proliferation and survival, DAP12 might be a substrate of DCIR-activated SHP-1 in the RANKL-activated PI3K/Akt pathway.

The PI3K/Akt-mediated signaling pathway is associated with RANKL-induced NFATc1. Overexpression of active form of Akt upregulated the expression of NFATc1. Akt induced phosphorylation of GSK3β, which is an inactive form of GSK3β, and the GSK3β is known as a potential regulator of NFATc1. Because active GSK3β reduces nuclear localization of NFATc1, phosphorylation of GSK3β (an inactive form) by Akt increased OC differentiation (28). Therefore, Akt activates osteoclastogenesis through regulating the expression and nuclear localization of NFATc1. Our previous study revealed that DCIR deficiency increased NFATc1 expression during osteoclastogenesis (18). Given the link of Akt with NFATc1, the upregulation of NFATc1 in *Dcir* ^−/−^ OCs may be induced by enhanced Akt activation. The future studies of DCIR-mediated signaling pathways will extend our understanding of the kinases and phosphatases-mediated regulation of osteoclastogenesis.

DCIR regulates gene expression related to the antigen-presentation ability of DCs and to pro-inflammatory status of vascular-resident macrophages (18, 34). RNA-seq analysis revealed that the gene sets associated with metabolic processes such as lipid metabolism and glycolysis were enriched in *Dcir* ^−/−^ OCs. These cells showed upregulation of the *Slc2a4* gene, which encodes GLUT4, an insulin-sensitive glucose transporter predominantly expressed in the adipose tissue and skeletal muscle. Glycolysis was increased during RANKL-induced OC differentiation, but glucose deprivation reduced the osteoclastic resorption rate but not differentiation, suggesting that glycolysis is an important energy source in bone resorption (35, 36). Lipids are another energy source, and different lipid species have distinct roles in OC differentiation and function (37, 38). Saturated fatty acids promote OC survival by preventing apoptosis, but their effects on OCs remains controversial (39). Cholesterol is an essential component of lipid bilayers and plays an indispensable role in numerous cell functions. Higher cholesterol levels may stimulate bone turnover because cholesterol-lowering statin drugs suppress osteoclastogenesis but increase bone formation (40). However, the relationship between statins and bone metabolism remains inconclusive. Interestingly, Park et al. showed that DCIR deletion downregulated genes related to lipid metabolism and that DCIR regulated cholesterol homeostasis in vascular macrophages (34). Further analyses of the *Dcir* ^−/−^ OC gene sets associated with glycolysis and lipid metabolism will provide novel insights into the metabolic processes in OCs and a deeper understanding of how these processes contribute to the treatment of human bone disorders.

In summary, we demonstrated that DCIR deficiency increased the responsiveness of BMMs to M-CSF and RANKL, which are essential factors for osteoclastogenesis. The interaction of DCIR with its endogenous ligand, NA2, downregulated Akt phosphorylation downstream of CSF-1R and RANK in BMMs, and DCIR-mediated signaling regulated gene expression in OCs. DCIR deficiency alters the gene expression pattern in OCs, and DCIR-mediated signaling might be involved in metabolic processes, specifically lipid biosynthesis and glycolysis. However, further analyses are needed to clarify the roles of neuraminidases in the regulation of terminal sugar moieties in N-glycans. Our previous study showed that the DCIR–NA2 axis regulated human OC differentiation, suggesting that DCIR agonists could be a therapeutic strategy for treating bone diseases caused by OCs.

## Materials and methods section

### Mice

*Dcir* ^−/−^ mice were generated as previously described (15) and were backcrossed to C57BL/6J at 12 generations. All the mice used in these experiments were 8–12-week old, and age- and sex-matched control C57BL/6J mice were purchased from Japan SLC (Shizuoka, Japan). The control C57BL/6J mice that were purchased from SLC were reared in the same mouse rack with *Dcir* ^−/−^ mice more than 1 week before using experiments. All mice were housed in specific pathogen–free animal rooms at the Research Institute for Biomedical Sciences, Tokyo University of Science, and at Tohoku Medical and Pharmaceutical University. Animal experiments were carried out following the guidelines of each university and were approved by the Animal Experimental Committee of the Tokyo University of Science and the Animal Experiments Committee of Tohoku Medical and Pharmaceutical University.

### *In vitro* culture of BM-derived macrophages and osteoclasts

To differentiate BM-derived macrophages, BMCs were isolated by flushing the BM cavities of femurs with α-minimal essential medium (Gibco, Life Technologies, CA) supplemented with penicillin (100 units/ml), streptomycin (100 μg/ml), and 10% heat-inactivated fetal bovine serum. Red blood cells were destroyed in hemolysis buffer (140 mM NH_4_Cl and 17 mM Tris-HCl, pH 7.2) for 10 min on ice, and the treated cells were pre-incubated in a 100-mm dish for 1 h. The non-adherent cells (hematopoietic cells) were harvested and seeded at 50,000 cells per well in 96-well plates in the presence of 20 ng/ml of recombinant human M-CSF (R&D Systems, MN) for 2 days. The cells that proliferated in response to M-CSF are defined as BMMs in this paper. To generate OCs, the BMMs were further cultured in the presence of 20 ng/ml of M-CSF and 100 ng/ml of human recombinant soluble RANKL (Oriental Yeast, Tokyo, Japan), with the culture medium changed every 2 days. To induce macrophage maturation, BMMs were incubated in 20 ng/ml of M-CSF for 6 days, with the medium changed every 2 days.

### Flow cytometry analysis

BMCs underwent blood cell lysis with hemolysis buffer on ice. The Fc receptors on these cells were blocked with 2.4G2, an Fc receptor blocker, at 4°C for 10 min. The cells were washed twice with a fluorescence-activated cell–sorting buffer consisting of 2% fetal bovine serum in phosphate-buffered saline (PBS) and incubated with fluorescence-conjugated antibodies for 30 min on ice. Commercially available antibodies used in the flow cytometry analysis are described in supplemental resource **Table S2**. Flow cytometry was performed on a FACSCanto II (Becton Dickinson, San Jose, CA) and analyzed using FlowJo software (Tree Star, Ashland, OR, USA).

### Tritium thymidine incorporation assay

BMMs were harvested using Cell Dissociation Solution Non-enzymatic 1× (Sigma-Aldrich,MO) and plated at 300,000 cells per well in 96-well plates in the presence of various concentrations of M-CSF. After 2–3 h, 1.0 μCi of [^3^H] thymidine (PerkinElmer, MA) was added and BMMs were further incubated at 37°C for 48 h. The cells were passed through glass fiber filters with the Skatron microplate washer supernatant collection system (Molecular Devices, CA) and the incorporation of [^3^H] thymidine into DNA was determined with the MicroBeta microplate counter system (PerkinElmer).

### Pit formation

OCs were generated in a 5-day culture in a 100-mm dish as described in the Materials and Methods section. The OCs were enzymatically harvested and reseeded at a concentration of 20,000 cells per well in an Osteo Assay plate (Corning, NY) in the presence of M-CSF and RANKL. After 3 days, the cells were removed by ultrasonic destruction for 30 s in a 1-M ammonia solution. The entire surface of each calcium phosphate–coated well was imaged by light microscopy (KEYENCE, Osaka, Japan), and the images were analyzed by ImageJ to examine the total resorbed area.

### Apoptotic assay

BMMs cultured for 2 days were incubated in M-CSF in combination with 100 ng/ml RANKL on 48-well plates at 10,000 cells per well. The medium was changed every 2 days. On days 7 and 8 after the beginning of BMC culture, the culture medium was carefully removed and cell lysates were prepared with a cell lysis buffer (500 μl) provided by Cell Death Detection ELISA (Roche Molecular Biochemicals, Mannheim, Germany) at room temperature (RT) for 30 min. As a positive control of cell apoptosis, all cytokines were removed from the wells during the last 6 h on day 8 of culture. Twenty microliters of the supernatant were analyzed to determine the magnitude of apoptosis by ELISA.

### RANKL-induced bone destruction and serum collection

Seven-week-old WT and *Dcir* ^−/−^ male mice were intraperitoneally administered saline or 100 μl of GST-RANKL (Oriental) for 3 days at 24-h intervals (41). Mice were sacrificed 1.5 h after the last injection of PBS or RANKL, and whole blood was obtained by cardiac puncture under anesthesia. The blood samples were left to clot at 4°C overnight and centrifuged at 3,000 rpm for 10 min to separate serum from coagulated blood. The serum was collected and stored at −80°C until assayed. The serum level of calcium ion was determined with the Calcium E-HA test (Wako, Osaka, Japan) by measuring the absorbance at 570 nm with a reference wavelength of 650 nm.

### Quantitative PCR

Total RNA was extracted from BMMs and OCs using the GenEluteTM Mammalian Total RNA Miniprep kit (Sigma-Aldrich). Quantification of total RNA was performed using NanoDrop (ThermoFisher SCIENTIFIC, MA). One microgram of total RNA was reverse-transcribed using Superscript II Reverse Transcriptase (Invitrogen, ThermoFisher SCIENTIFIC) to synthesize the initial cDNA. qPCR was carried out in a total reaction volume of 10 μL that included 5 ng equivalent to total RNA, TB Green Premix Ex TaqII (Takara Bio, Kusatsu, Japan), and a set of forward and reverse primers (Operon, Germany) in duplicate or triplicate samples. Quantification was performed on a CFX96™ Real-Time system (Bio-Rad Laboratories, Hercules, CA). The cycling program involved 1 cycle at 95°C for 1 min, followed by 44 cycles at 95°C for 3 s and 60°C for 30 s. The primer sets used to detect each transcript in macrophages or OCs are described in supplemental resource **Table S1**. The final concentration of the primers was 800 nM. GAPDH was used as an internal housekeeping gene control. The target genes and housekeeping gene were quantified simultaneously in one plate, with water samples as the negative reference. Sample differences in RNA quality and initial quantity were normalized to GAPDH. The relative expression of the targeted gene (2^^-ddCt^ method) was calculated from the following equation: 2^^-dCt^, where dCt = average Ct for the gene of interest − average Ct for the housekeeping gene (42, 43).

### Biochemical analysis

For western blotting of M-CSF– and RANKL-mediated signals, BMMs were re-plated in 12-well plates at 250,000 cells per well in the presence of 5 ng/ml of M-CSF. After 24-h culture, the BMMs were incubated in serum-free medium without M-CSF but with NA2 (1 μg/ml) for 6 h before stimulation. The cells were activated with 20 ng/ml of M-CSF or 100 ng/ml of RANKL in the presence of NA2 for the periods described in the experiments. The cells were lysed to prepare cell lysates in 1% NP-40 (consisting of 10 mM Tris-HCl, pH 7.5, 100 mM NaCl, 5 mM MgCl_2_ with proteinase inhibitor cocktail (Thermo Fisher Scientific, Waltham, MA, USA) and phosphatase inhibitor cocktail (Roche)). The lysates were kept on ice for 10 min and clarified by centrifugation at 15,000 rpm for 10 min. Equal aliquots of lysates were loaded in SDS-polyacrylamide gels and separated at 230 V for 30 min. Each gel was electrophoretically transferred onto one PVDF membrane at a constant 230 mA for 25 min per membrane in a semidry system (Bio-Rad). The membranes were blocked by 5% bovine serum albumin (Sigma-Aldrich) in Tris-buffered saline containing 5% Tween 20 for 1 h at RT and incubated with primary antibodies at 4°C overnight, followed by probing with horseradish peroxidase–conjugated secondary antibodies. The primary antibodies used in this biochemical assay are described in supplemental resource **Table S3**. The membrane was subjected to the ECL Prime Western Blotting Detection System (GE Healthcare UK, Amersham, England) to visualize protein signals. To relatively quantify protein bands of western blots, we used ImageJ freeware from National Institute of Health (MD). We calculated the relative amounts as a ratio of phosphorylated protein band compared to loaded unphosphorylated protein.

### RNA-seq analysis

The differentiation of OCs was induced in the presence of M-CSF and RANKL for 4 days, and RNA samples were extracted using the FastGene™ RNA basic kit (Nippon Genetics). The RNA quality was assessed by RNA Screen Tape using a TapeStation System (Agilent Technologies, CA). High-quality RNA samples with an RNA integrity number greater than 7.0 were used for RNA library construction. RNA-seq library construction and subsequent next-generation sequencing were conducted by Macrogen Japan Corp. (Tokyo, Japan). Briefly, total RNA was quantified by the Quant-IT RiboGreen RNA Assay (Invitrogen) and qualified using the TapeStation system. One milligram of total RNA per sample was prepared using the Illumina TruSeq Stranded mRNA Sample Prep Kit (Illumina, San Diego, CA, USA). mRNA containing poly-A was enriched by poly-T–attached magnetic beads, and the mRNA samples were fragmented with divalent cations under elevated temperature. The fragmented RNAs were reverse transcribed for the first strand of cDNA with SuperScript II reverse transcriptase (Invitrogen) using random primers, and for the second strand with DNA polymerase I, RNase H, and dUTP. The 5’ and 3’ ends of the fragmented RNA were repaired, and indexed adaptors were added *via* ligation. The products were purified and enriched by PCR to create the final cDNA library. The generated libraries were quantified using KAPA Library Quantification kits for Illumina Sequencing platforms according to the qPCR Quantification Protocol Guide (Kapa Biosystems), and the fragment size was assayed using D1000 DNA Screen Tape in TapeStation. Libraries with unique sample barcodes were pooled and sequenced using an Illumina NovaSeq (Illumina), and the paired-end (2×100 bp) sequencing was performed by Macrogen Japan Corp.

### RNA-seq data processing

The raw sequencing data were processed using TrimGalore v0.6.6 to remove adapter sequences and low-quality reads. The processed reads were used to align to the mouse genome reference mm10 using hisat2 v2.2.0. Aligned read counts that fell on the exons of each gene were calculated by the R package Rsubread v1.32.4. The read counts were normalized and compared for differential gene expression using the R package edgeR v3.24.3. The RNA-seq data have been deposited in the Gene Expression Omnibus database under accession no. GSE223429.

### Functional signatures of changes in OC gene expression

GO enrichment analysis was performed using the PANTHER Overrepresentation Test (Released 20200728) to identify functional pathways. Differentially expressed genes with |Fold Change| > 0.5 were selected for the analysis. The functional signatures of gene sets were re-categorized as follows: genes annotated with the MHCII protein complex, antigen processing and presentation, and the regulation of T cell proliferation were categorized as being related to antigen presentation and T cell proliferation. GSEA was conducted by javaGSEA software using WikiPathways as gene sets.

### Statistics

The statistical significance between groups was determined using the two-tailed unpaired Student’s *t*-test (*P<0.05; **P<0.01).

## Data availability statement

The datasets presented in this study can be found in online repositories. The names of the repository/repositories and accession number(s) can be found below: GSE223429 (GEO).

## Ethics statement

The animal study was reviewed and approved by The Animal Experimental Committee of the Tokyo University of Science and the Animal Experiments Committee of Tohoku Medical and Pharmaceutical University.

## Author contributions

TK conducted most of the experiments; TK and TM analyzed DCIR signaling mechanisms; KS performed RNA-seq analysis; S-HC prepared cellular samples; TK wrote the draft manuscript; TK, AN, and YI organized and supervised the project and edited the draft manuscript. All authors contributed to the article and approved the submitted version.

## Supplementary material

Supplementary Figure 1 DCIR deficiency does not affect cell survival of *Dcir* ^−/−^BMMs. **(A)** Apoptotic cell analysis. WT and *Dcir* ^−/−^ BMMs were induced to form OCs for 7 and 8 days. As a positive control of apoptosis, at 8 days, the medium containing M-CSF and RANKL was replaced for 6 h with medium lacking both cytokines. Mono- and oligonucleosomes in the cytoplasmic fraction of cell lysates were detected using the Cell Death Detection ELISA kit. Data are representative of two independent experiments. The bars show the means ± s.d. of triplicate cultures.

Supplementary Figure 2 DCIR deficiency has no effect on osteoclast precursor proportion in BM. **(A)** Flow cytometric analysis of the OC precursor population in 8-week-old WT and *Dcir* ^−/−^ mice. BMCs negative for CD3, B220, and Ter119 were plotted for CD11b and Ly6C. The OC precursor population is characterized by CD11b^lo^Ly6C^hi^. The dot plots represent the data from three mice. **(B)** The frequency of three distinct populations in WT and *Dcir* ^−/−^ BMCs. Data are expressed as means ± s.d. of three mice and are representative of two independent experiments **(A, B)**. **(C)** BMCs were plotted for CD265 and CD115. **(D)** The percentage of three distinct populations in WT and *Dcir* ^−/−^ BMCs. **(E)** CD11b^-/low^, CD3^-^, and B220^-^ (triplicate negative) BMCs were plotted for CD117 and CD115. The OC precursor populations are characterized by CD117^hi^, CD117^int^, and CD117^low^ in CD115–positive BMCs. **(F)**. The frequency of three distinct populations in WT and *Dcir* ^−/−^ BMCs. The dot plots were shown as a representative data of two independent experiments. Data are means ± s.d. of triplicate wells **(C–F)**.

Supplementary Figure 3 DCIR deficiency affects the gene expression profile of osteoclasts. **(A)** Heatmap of gene changes associated with osteoclastogenesis. **(B)** Heatmap of changes in the gene set associated with the category related to antigen presentation and T cell proliferation. The gene sets were selected according to our previous study (18). **(C)** Heatmap of gene changes associated with neuraminidases and sialyltransferases. Gene expression was analyzed in WT (n=3) and *Dcir* ^−/−^ (n=3) OCs. The gene sets of the indicated categories are represented by a color scale from high (orange) to low (blue).
