# Supplementary figures and images for: DCIR suppresses osteoclastic proliferation and resorption by downregulating M-CSF and RANKL signaling
Source: Front Immunol. 2023 May 17;14:1159058. doi: 10.3389/fimmu.2023.1159058 (PMC10230091; doi:10.3389/fimmu.2023.1159058)

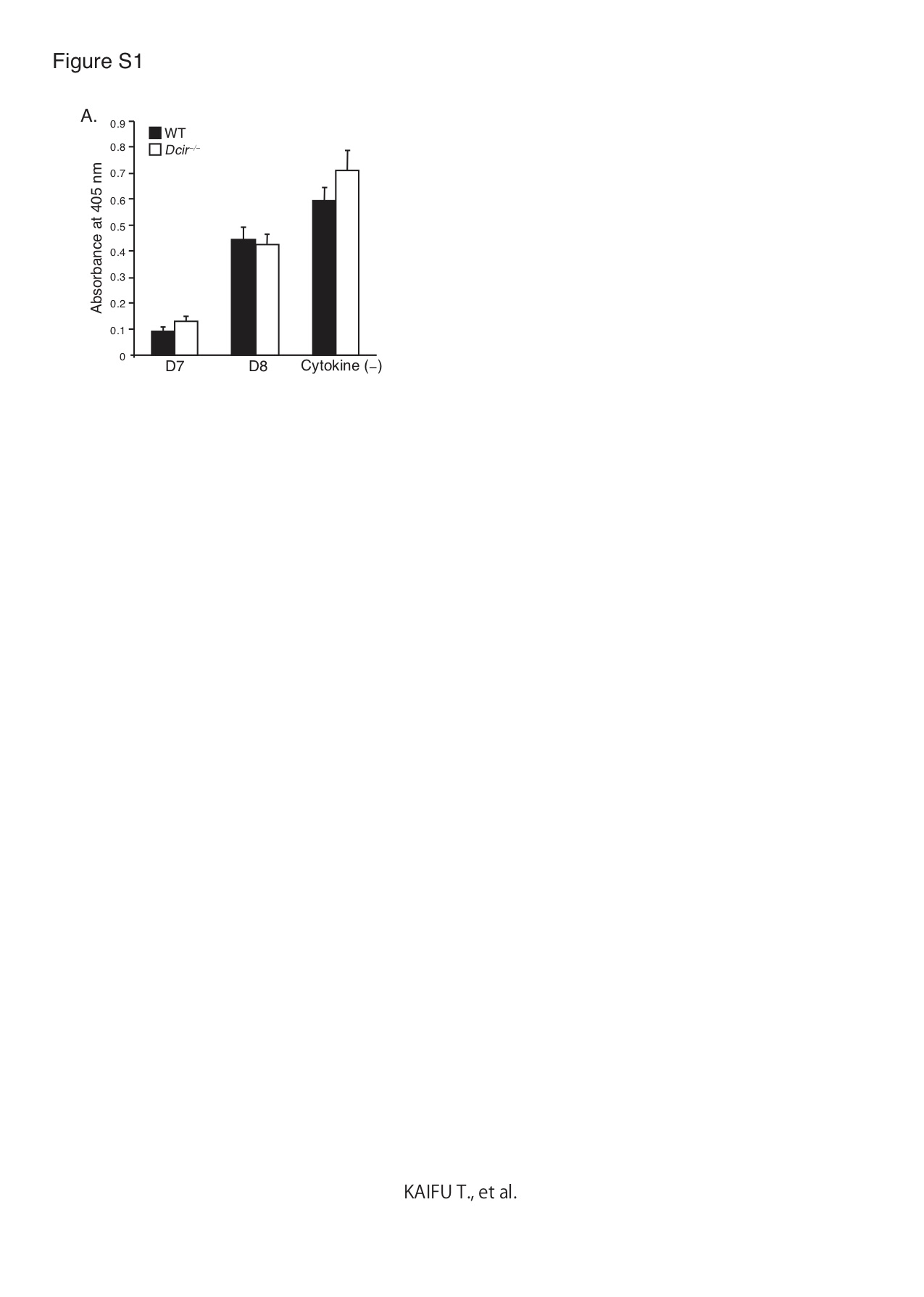

Supplement: Supplementary Figure 1 — DCIR deficiency does not affect cell survival of Dcir −/−BMMs. (A) Apoptotic cell analysis. WT and Dcir −/− BMMs were induced to form OCs for 7 and 8 days. As a positive control of apoptosis, at 8 days, the medium containing M-CSF and RANKL was replaced for 6 h with medium lacking both cytokines. Mono- and oligonucleosomes in the cytoplasmic fraction of cell lysates were detected using the Cell Death Detection ELISA kit. Data are representative of two independent experiments. The bars show the means ± s.d. of triplicate cultures. [file Image_1.jpg]

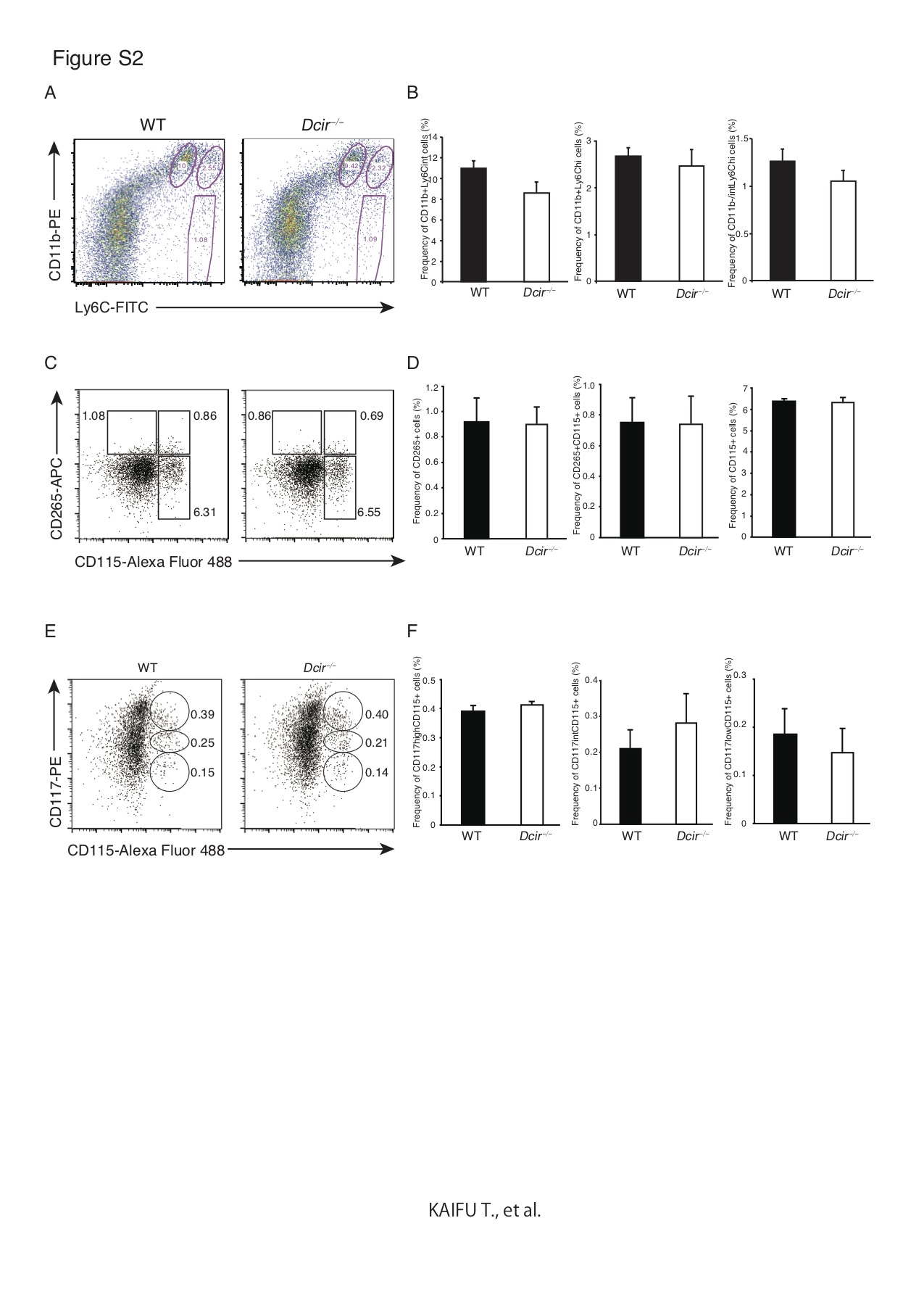

Supplement: Supplementary Figure 2 — DCIR deficiency has no effect on osteoclast precursor proportion in BM. (A) Flow cytometric analysis of the OC precursor population in 8-week-old WT and Dcir −/− mice. BMCs negative for CD3, B220, and Ter119 were plotted for CD11b and Ly6C. The OC precursor population is characterized by CD11bloLy6Chi. The dot plots represent the data from three mice. (B) The frequency of three distinct populations in WT and Dcir −/− BMCs. Data are expressed as means ± s.d. of three mice and are representative of two independent experiments (A, B). (C) BMCs were plotted for CD265 and CD115. (D) The percentage of three distinct populations in WT and Dcir −/− BMCs. (E) CD11b-/low, CD3-, and B220- (triplicate negative) BMCs were plotted for CD117 and CD115. The OC precursor populations are characterized by CD117hi, CD117int, and CD117low in CD115–positive BMCs. (F). The frequency of three distinct populations in WT and Dcir −/− BMCs. The dot plots were shown as a representative data of two independent experiments. Data are means ± s.d. of triplicate wells (C–F). [file Image_2.jpg]

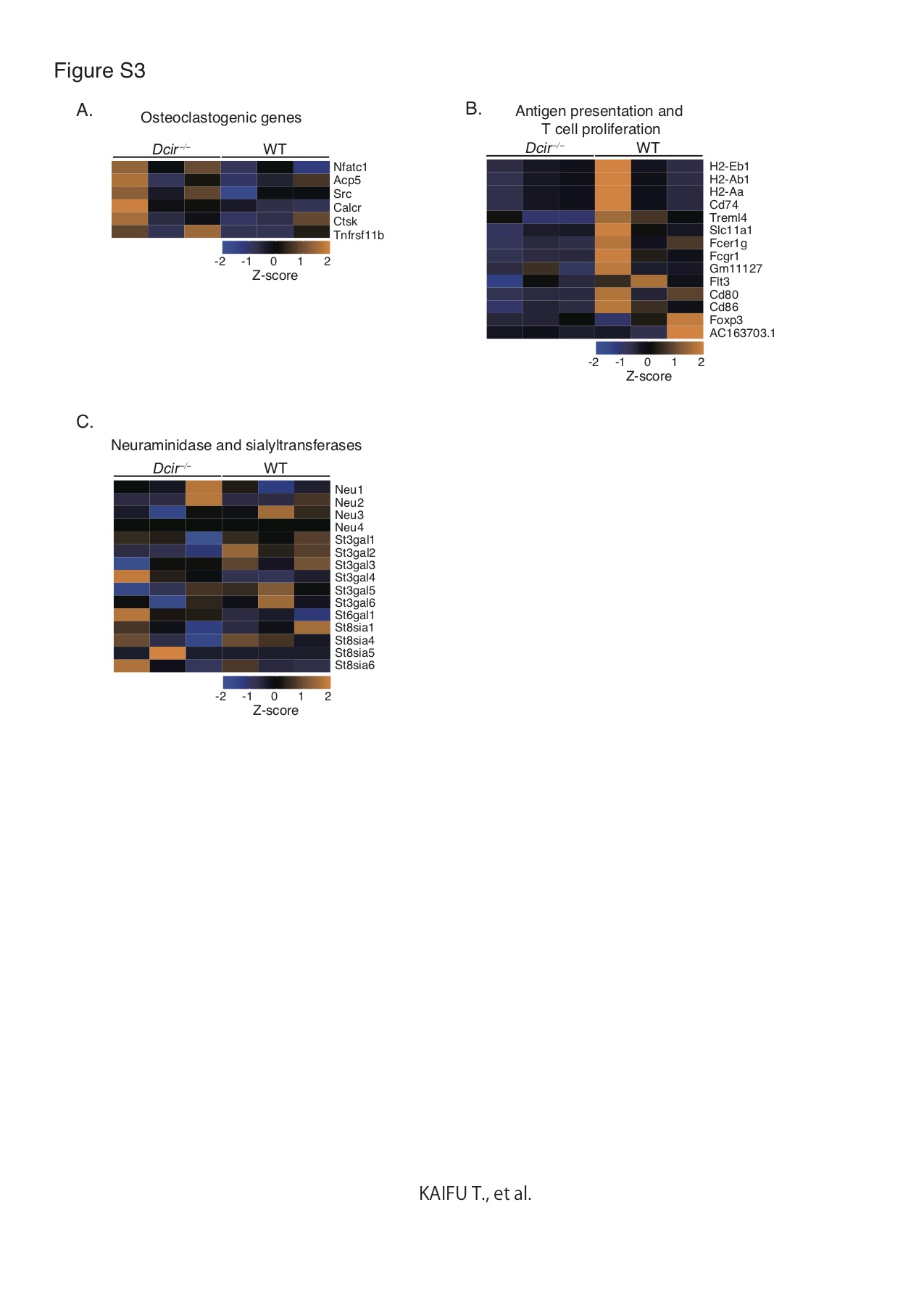

Supplement: Supplementary Figure 3 — DCIR deficiency affects the gene expression profile of osteoclasts. (A) Heatmap of gene changes associated with osteoclastogenesis. (B) Heatmap of changes in the gene set associated with the category related to antigen presentation and T cell proliferation. The gene sets were selected according to our previous study (18). (C) Heatmap of gene changes associated with neuraminidases and sialyltransferases. Gene expression was analyzed in WT (n=3) and Dcir −/− (n=3) OCs. The gene sets of the indicated categories are represented by a color scale from high (orange) to low (blue). [file Image_3.jpg]
