# Supplementary material for: DCIR suppresses osteoclastic proliferation and resorption by downregulating M-CSF and RANKL signaling
Source: Front Immunol. 2023 May 17;14:1159058. doi: 10.3389/fimmu.2023.1159058 (PMC10230091; doi:10.3389/fimmu.2023.1159058)
Supplement: Supplementary file 4 [file Table_1.docx]

Supplementary information

**DCIR suppresses osteoclastic proliferation and resorption by downregulating M-CSF and RANKL signaling**

**Running title: DCIR - mediated inhibition of M-CSF and RANKL signaling**

Tomonori Kaifu, Takumi Maruhashi, Soo-Hyun Chung, Akira Nakamura, and Yoichiro Iwakura

**Supplemental Table S1. List of antibodies**

**Antibodies Label Clone Source Catalog # Identifier**

**For FACS analysis**

Anti-mouse CD11b PE M1/70 BioLegend 101207 AB_312790

Anti-mouse Ly6C FITC HK1.4 BioLegend 128005 AB_1186134

Anti-mouse CD3 Pacific Blue 17A2 BioLegend 100213 AB_493644

Anti-mouse B220 PerCP/Cy5.5 RA3-6B2 BioLegend 103235 AB_893356

Anti-mouse CD11b PE/Cy7 M1/70 BioLegend 101216 AB_312799

Anti-mouse CD265 APC R12-31 BioLegend 119807 AB_2892276

Anti-mouse CD115 Alexa Fluor 488 AFS98 BioLegend 135511 AB_11218605

Anti-mouse CD117 PE 2B8 BioLegend 105807 AB_313216

**Supplemental Table S2 PCR primer sets**

**Gene Forward (5' to 3') Reverse (3' to 5')**

Neu1 GGACCGCTGAGCTATTGGG CGGGATGCGGAAAGTGTCTA

Neu2 CACAGGCGTCCATGCTTACA CTGCGTGCTCATCCGTCTT

Neu3 AGCAGGAAGAACAGAGTGGG CCTCCATCAGTAGCCGTTGG

Neu4 CGTGTGCCTGCGTTACTCT CAAGGGTTCATAGACCTGTGC

**Supplemental Table S3. List of antibodies**

**Antibodies Specie Source Catalog #**

**For WB analysis**

Akt Rabbit Cell signaling technology 4691

Phospho-Akt(Ser473) Rabbit Cell signaling technology 9271

p44/42MAPK Rabbit Cell signaling technology 4695

Phospho- 44/42MAPK Rabbit Cell signaling technology 4370

(Thr202/Tyr204)

p38 MAPK Rabbit Cell signaling technology 8690

Phospho-p38 Rabbit Cell signaling technology 4511

(Thr180/yr182)

IκBα Mouse Cell signaling technology 4814

Phospho-IκBα(Ser32) Rabbit Cell signaling technology 2859

β-actin Mouse Sigma-Aldrich A1978
